# Clinico-pathological associations and concomitant mutations of the RAS/RAF pathway in metastatic colorectal cancer

**DOI:** 10.1186/s12967-019-1879-2

**Published:** 2019-04-29

**Authors:** Edoardo Isnaldi, Anna Garuti, Gabriella Cirmena, Stefano Scabini, Edoardo Rimini, Lorenzo Ferrando, Michela Lia, Roberto Murialdo, Lucia Tixi, Enrico Carminati, Andrea Panaro, Maurizio Gallo, Federica Grillo, Luca Mastracci, Lazzaro Repetto, Roberto Fiocca, Emanuele Romairone, Gabriele Zoppoli, Alberto Ballestrero

**Affiliations:** 1 Department of Internal Medicine (Di.M.I.), University of Genoa, Viale Benedetto XV, 6, 16132 Genoa, Italy; 2 Department of Integrated Surgical and Diagnostic Sciences (DISC), University of Genoa, Genoa, Italy; 3 Ospedale Policlinico San Martino IRCCS Per l’Oncologia, Genoa, Italy; 4 Department of Oncology, Ospedale Civile “G Borea”, Sanremo, Italy

**Keywords:** Metastatic colorectal cancer, Extended RAS, RAS/RAF pathway, Anti-EGFR, Concomitant mutations

## Abstract

**Background:**

Over the past few years, next-generation sequencing (NGS) has become reliable and cost-effective, and its use in clinical practice has become a reality. A relevant role for NGS is the prediction of response to anti-EGFR agents in metastatic colorectal cancer (mCRC), where multiple exons from KRAS, NRAS, and BRAF must be sequenced simultaneously.

**Methods:**

We optimized a 14-amplicon NGS panel to assess, in a consecutive cohort of 219 patients affected by mCRC, the presence and clinico-pathological associations of mutations in the KRAS, NRAS, BRAF, and PIK3CA genes from formalin-fixed, paraffin-embedded specimens collected for diagnostics and research at the time of diagnosis.

**Results:**

We observed a statistically significant association of RAS mutations with sex, young age, and tumor site. We demonstrated that concomitant mutations in the RAS/RAF pathway are not infrequent in mCRC, and as anticipated by whole-genome studies, RAS and PIK3CA tend to be concurrently mutated. We corroborated the association of BRAF mutations in right mCRC tumors with microsatellite instability. We established tumor side as prognostic parameter independently of mutational status.

**Conclusions:**

To our knowledge, this is the first monocentric, consecutively accrued clinical mCRC cancer cohort tested by NGS in a real-world context for KRAS, NRAS, BRAF, and PIK3CA. Our study has highlighted in clinical practice findings such as the concomitance of mutations in the RAS/RAF pathway, the presence of multiple mutations in single gene, the co-occurrence of RAS and PIK3CA mutations, the prognostic value of tumor side and possible associations of sex with specific mutations.

**Electronic supplementary material:**

The online version of this article (10.1186/s12967-019-1879-2) contains supplementary material, which is available to authorized users.

## Background

In recent years, major advances have been made in the treatment of metastatic colorectal cancer (mCRC) thanks to the introduction of novel therapies, such as monoclonal antibodies (moAbs), which are designed against specific molecular targets [1]. Two such moAbs, cetuximab and panitumumab, which target the epidermal growth factor receptor (EGFR), have demonstrated their efficacy in a subgroup of patients with mCRC characterized by specific molecular aberrations. However, only a small subgroup of patients (10% to 20%) achieves a response to anti-EGFR moAb single-agent treatment [2]. This poor response rate can be explained, in part, by activating mutations in signaling pathways downstream EGFR, such as in the RAS/RAF pathway [3]. Primary anti-EGFR resistance factors were first observed in patients harboring mutations in the genes encoding KRAS exon 2 [4]. Other primary anti-EGFR resistance factors have since been described, including additional mutations in KRAS (at exons 3, 4) and NRAS (at exons 2, 3, and 4) [5]. Likewise, mutations in the BRAF gene have been described as a prognostic marker and in some works as a predictive factor for resistance to anti-EGFR moAb [6]. In addition, PIK3CA mutations in exon 20 have been associated with resistance to anti-EGFR treatment in patients with wild-type KRAS [7]. However, as mutations in PIK3CA are often associated with mutations in KRAS, the role of PIK3CA as an independent predictive marker of response to anti-EGFR needs to be confirmed.

Next-generation sequencing (NGS) can analyze several genes simultaneously, at lower cost, person-time, and higher sensitivity than traditional, capillary-based sequencing methods. NGS has been retrospectively used to evaluate response to anti-EGFR therapies in mCRC patients [8]. In this study, we used a custom-designed 14-amplicon NGS panel to assess the presence of mutations in the KRAS, NRAS, BRAF, and PIK3CA genes in a single-center cohort of 219 consecutive patients affected by mCRC. We also investigated the clinico-pathological implications of those mutations and their prognostic impact in our cohort of patients.

## Methods

### NGS panel design and sequencing

Somatic hotspots in KRAS, NRAS, BRAF, and PIK3CA, known to be associated with mCRC, were obtained from COSMIC v69, and used as an input for the Ion AmpliSeq Designer™ online tool. Overall, 14 multiplexed primer pairs spanning 1556 base pairs of genomic DNA were designed and used, after titration experiments using two mCRC samples known to be KRAS mutated and wild types, respectively. One hundred pre-stored consecutive samples with known mutations in the aforementioned genes, previously assessed by capillary sequencing as part of our routine diagnostics were resequenced by NGS on Ion Torrent™ 314™ chips, multiplexing an average of 8 samples per chip using AmpliSeq™ barcode kits. The following one hundred nineteen samples were collected consecutively and analyzed by NGS. Sensitivity and specificity of our platform, when sequencing at depth in excess of 1000×, were greater than 99% for FFPE samples with a mutant allelic fraction above 5%.

### Analyses and statistics

The analysis pipeline used was as follows: in brief, runs were analyzed on a Dell workstation using the Ion Torrent™ 3.4 alignment and calling pipeline, using hg19 as the reference genome. To increase specificity of our results, as per manufacturer’s instructions, we limited our analyses to target and hotspot regions defined by COSMIC v69 and provided as an output by the Ion AmpliSeq Designer™ tool (files available upon request to the corresponding author). The effective presence and deleteriousness of found mutations, when falling outside the known hotspots identified by COSMIC, was confirmed by manual inspection of the pileups using IGV, and subjected to functional prediction according to a majority consensus of SIFT, PolyPhen2, and MutationAssessor [9–11]. Mutational variables for downstream statistical analyses were selected using a well-established penalized maximum likelihood regression model implemented in the R package *glmnet* [12]. For the comparison of mutational and clinical variables, we employed multiple logistic regression corrected with the Firth method, adjusting for stage, age, anatomical site, and gender when appropriate. Pairwise associations between categorical variables were evaluated by Fisher’s exact test, while differences of continuous variables among two or more groups were assessed using Wilcoxon or Kruskal–Wallis tests, respectively.

In survival analyses, we considered as our primary endpoint progression-free survival upon beginning a first line of chemotherapy in the metastatic setting. Survival curves were plotted using the Kaplan–Meier estimators, generated with the package survcomp [13], and p-values were calculated using the log-rank test. For survival analyses including more than one variable, a stepwise backward–forward Cox proportional hazards regression model was employed, starting from all clinical and pathological variables described above, until minimization of the Akaike Information Criterion was achieved (package MASS) [14].

Multiple correspondence analysis (MCA) was performed using the package FactoMineR (http://factominer.free.fr/contact/index.html), after categorizing variables as described, and results were represented with ggplot2 [13]. The mutual exclusivity and lollipop plots in Fig. 4 were generated using the online tools OncoPrinter and MutationMapper [15, 16]. All statistical analyses were conducted within the R environment for statistical computing [17].

### Quantification of rare somatic cancer mutations using digital PCR

We validated using digital PCR (dPCR) assays co-occurring somatic mutations identified in KRAS (G12S, G12 V, G12I, G12C, G12F, G12D, G15S, G13C), NRAS (G13S), BRAF (S467L), PIK3CA (G1050S). The target selection was based on the prevalence of mutational co-occurrences in our set as well as on the commercial availability of pre-validated TaqMan^®^ SNP Genotyping Assays. These are single-tube assays containing dPCR primers and a probe for both the wild-type and mutant alleles. We prepared a reaction mix for each sample for two technical replicates (2 chips): 4 μL of DNA (20 ng/µL) with 11.7 μL nuclease-free water, 17.4 μL QuantStudio™ 3D Digital PCR Master Mix, 1.7 µL Custom TaqMan^®^ SNP Genotyping Assays (20×). The dPCR reaction was loaded onto a QuantStudio™ 3D Digital PCR Chip v2, and PCR was performed using the ProFlex 2× Flat PCR System with the program outlined in Additional file 1: Table S1. We analyzed the chips using the QuantStudio™ 3D Digital PCR Instrument and the QuantStudio^®^ 3D Analysis Suite™ Software.

## Results

### Patients

Over 24 months, 264 specimens from colorectal cancer (CRC) patients were brought to our laboratory for KRAS, NRAS, and BRAF characterization (see Additional file 1: Figure S1). Per protocol, PIK3CA hotspots regions were also assessed in the current study, but not reported to the oncologist in charge of the patient when bearing mutations of potential pathological significance. Of 264 samples that derived all from the primary tumors, 45 were not assessed because there was no indication for RAS testing (non-metastatic patients). Samples from 219 patients were then analyzed with NGS. No sample failed NGS testing, although we had to re-extract tumor DNA in 13 cases due to poor material quality, and we had to generate amplicon libraries twice in 10 cases because of experimental failures along the library preparation pipeline.

Patient characteristics are reported in Additional file 1: Table S2. Median age was 70 years (interquartile range 61–76), 52% patients of the analyzed cohort were male, and 69% of neoplasms with available stage data (n = 156) pertained to the left colon, with most patients presenting with a large (T3 or greater), node-positive tumor at first diagnosis. Microsatellite staining by immunohistochemistry was available for 113 patients of the total population and showed 9 patients (8% of the assessed cases) with loss of MLH1 (8 patients), MSH6 (1 patient), MSH2 (no patients). These demographic and anatomopathological features were well balanced according to sex, and in line with the epidemiologic characteristics of the mCRC patients diagnosed in our region. The relatively high median age is indeed in line with the patients referring to our hospital, which is situated in the region hosting the oldest population in Europe. Outcome data of patients were available for 101 patients of the entire population.

### Unbiased detection of clinically relevant mutations in clinical-practice NGS

Of 219 assessed samples analyzed, 143 (65%) presented at least one deleterious mutation in any of the four sequenced genes. The number of patients with mutations in a single gene was 109; with two mutations, 31; and with three mutations, 3. Mutations were not detected in any of the KRAS, NRAS, BRAF, and PIK3CA genes (quadruple mutation-negative patients) in 76 (35%) tumors.

**Fig. 1:**
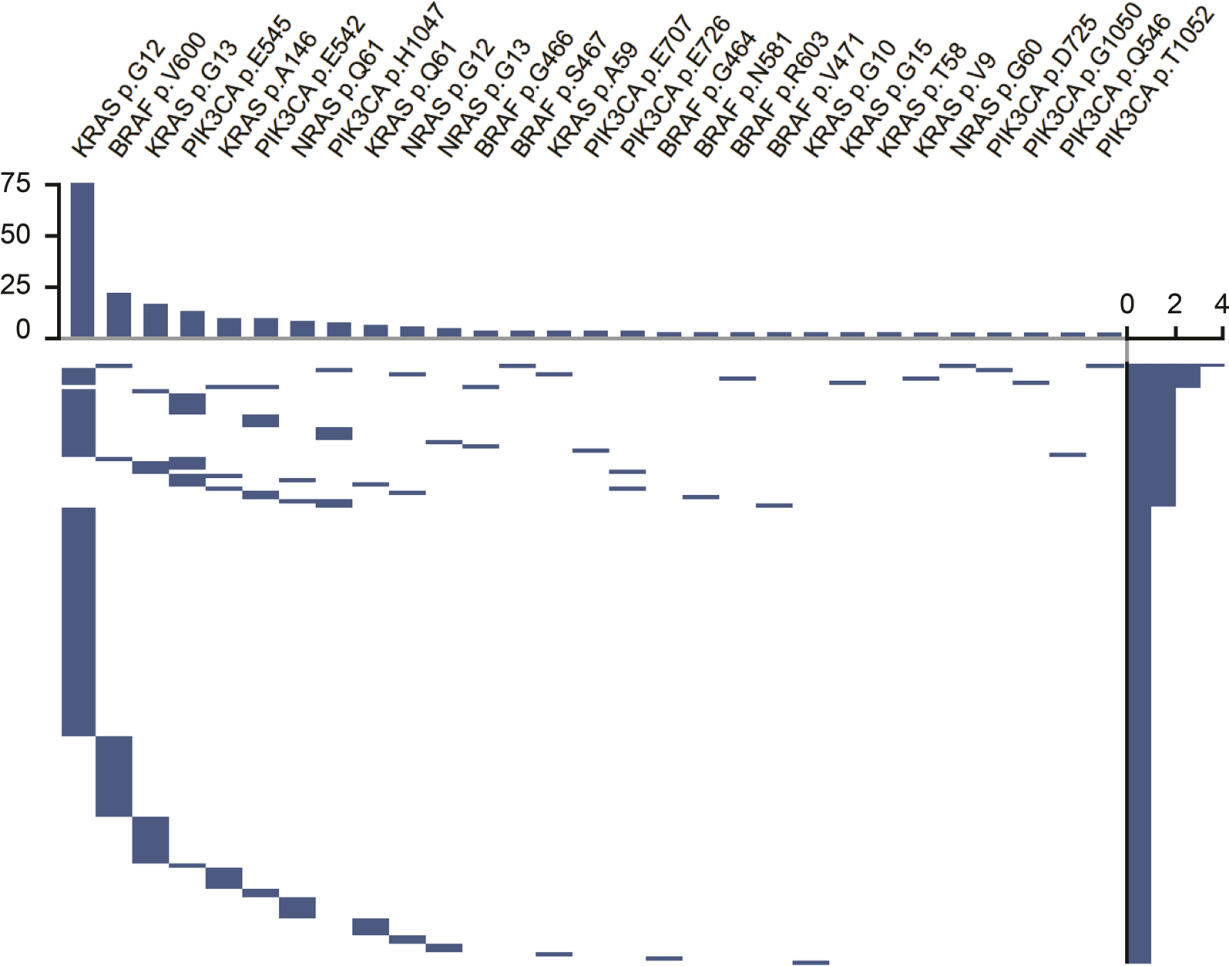
Map of type of mutations and their mutual relations. Top: bar plot of mutational frequency for each gene. Right side: bar plot of number of mutations for each patient. Main figure: mutual relationship of mutational events by patients

We identified 104 (47%) tumors with KRAS mutations, including one tumor with three KRAS p.G12C, p.G12V, and pG13C mutations and six tumors with two KRAS mutations p.G12V and p.G15S; pG12S and p.G12V; p.G12D and p.V9F; p.G12S and p.A59E; p.G12D and p.T58I; p.G12C and p.G13D. The mutations were located in exon 2 in 92 tumors (42%), exon 3 in 7 tumors (3.2%), and exon 4 in 8 tumors (3.6%). p.G12D, p.G12V, and p.G13D were the most common KRAS mutations in our cohort of colorectal cancers (see Fig. 1). Mutations located outside exon 2 were observed in 19 tumors (8%).

Fifteen tumors (7%) harbored a NRAS mutation. Seven mutations (3%) were located in exon 2 and 8 (4%) were located in exon 3. No mutations were found in exon 4. We found four NRAS p.G12C, p.G12D, p.G12V mutations in codon 12, three mutations p.G13D, p.G13R, p.G12S in codon 13, seven mutations p.Q61 K, p.Q61R in codon 61, and one mutation p.G60E in codon 60.

Twenty-eight tumors (13%) harbored a BRAF mutation including one tumor with two BRAF mutation p.S467L and p.V600M. Twenty-three mutations (10%) are located in exon 15 and six mutations (2.7%) were located in exon 11. p.V600E was the most common mutation for BRAF, since it was found in 20 tumors (9%). One tumor harbored an unusual p.V600M mutation with deleterious effect. Eight tumors (3.6%) harbored non-V600 mutations.

Thirty-three tumors (15%) harbored a PIK3CA mutation including one tumor with two PIK3CA mutations p.D725 N and p.H1047Y. Twenty mutations were located in exon 9 (9%), 8 mutations in exon 20 (3.6%), and 6 mutations in exon 13 (2.7%). Three codons (p.E542, p.E545, and p.H1047) account for 26 of 33 PIK3CA mutations (78%). Only three tumors (1.3%) were characterized by a single PIK3CA mutation without any concomitant alterations in the assessed genes.

### Associations between genetic alterations and biological features

**Fig. 2:**
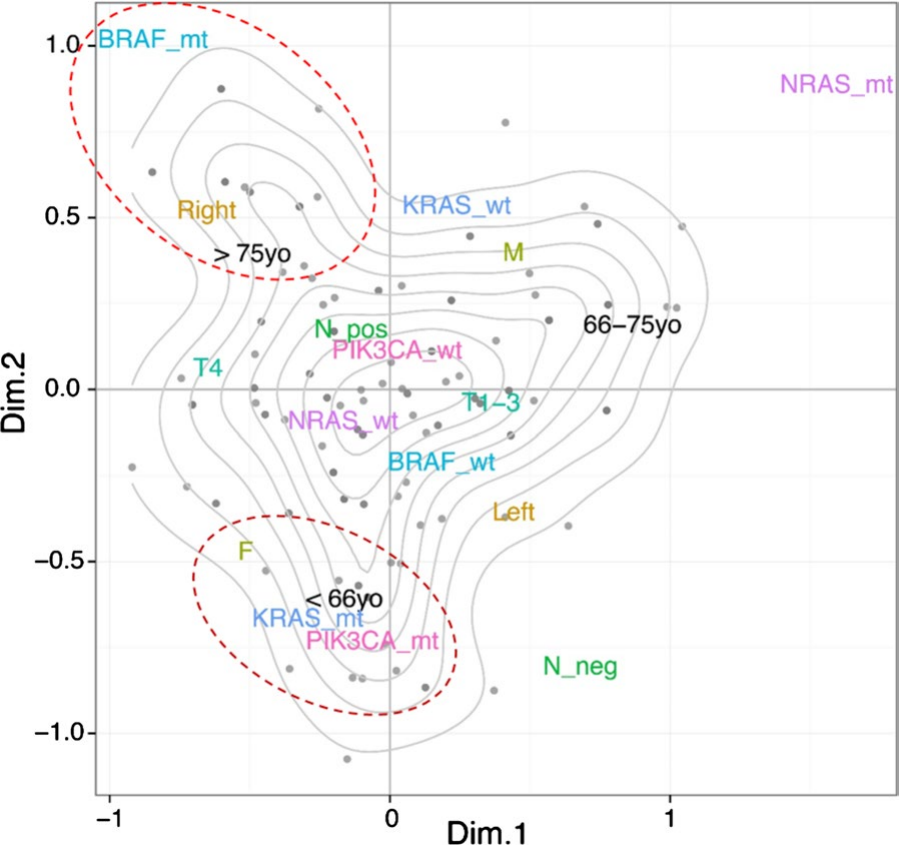
Multiple correspondence analysis (MCA) defines underlying the structure of clinical and pathological associations in the KRAS, NRAS, BRAF, and PIK3CA genes in mCRC. x- and y-axes represent the first and second dimension (Dim.1 and Dim.2) of the MCA analysis performed on clinical and pathological data from 219 mCRC patients collected in our center. In particular, we found a statistically significant association of RAS mutations with sex, young age and tumor site (lower left region) and BRAF mutations with anatomical site and old age (upper left region)

Sixty-eight (31%) tumors were located in the right colon and 151 (69%) tumors were located in the left colon. Quadruple mutation-negative tumors were observed in 13 right-sided colorectal cancers compared to 49 left-sided colorectal cancers (19 vs 41%, p = 0.001). Right-sided colorectal cancers have had a significantly higher incidence of mutations in BRAF (Additional file 1: Table S3, 8% vs 5%, p = 0.0001). Even though MSI staining was available in 113 of 219 patients, we found 7 patients (6%) with BRAF mutations and MSI status. Tumors with BRAF mutations were more frequently associated with microsatellite instability (6% vs. 2%, p = 0.0009). KRAS mutations were more frequent in females than in males (26% vs. 22%, p = 0.0460), as were BRAF mutations (8% vs 5%, p = 0.0269). In order to explore in an unbiased way the associations of the RAS/RAF pathway with clinical and pathological variables in colorectal cancer, i.e., stage, tumor site, nodal status, sex, and age, we used multiple correspondence analysis (MCA). MCA is a multivariate statistical method akin to principal component analysis but suited for categorical data; as a result, we reduced our variables of interest to two dimensions, which explain the largest fraction of the variance observed in our data set. Clinical and pathological variables together with KRAS, NRAS, BRAF, and PIK3CA status were projected as vectors in a space defined by those two dimensions (see Fig. 2). The position of the variable categories in this two-dimensional space reflects their mutual associations, with no a priori assumption on the underlying structure of the data. We observed that tumors with BRAF mutations fell in proximity of “older age” and “right colon side”. In contrast, RAS mutations clustered with PIK3CA mutations, “younger age”, and “female sex”. In summary, by both MCA analysis and classic statistical tests we could observe two groups of patients enriched in specific mutational and clinico-pathological features (“BRAF mutations”, “right side”, “old age” in group 1 and “RAS mutations”, “PIK3CA mutations”, “younger age”, and “female sex” patients in group 2).

### Coexistence of mutations in mutually exclusive genes revealed by clinical practice NGS

We found 34 tumors (15%) with concomitant mutations within KRAS, NRAS, BRAF, and PIK3CA genes. Thirty-one tumors presented concomitant mutations in two genes; three tumors presented concomitant mutations in three genes including one tumor with BRAF, NRAS and PIK3CA concomitant mutations and two tumors with BRAF, KRAS, and PIK3CA concomitant mutations. Concomitant KRAS mutations were observed in 23 of 33 PIK3CA-mutated tumors (63%), 2 of 15 NRAS-mutated tumors (13%), and 4 of 29 BRAF mutated tumors (13%). Tumors with a PIK3CA mutation showed a significantly higher incidence of concomitant mutations (90%) as compared with tumors with KRAS (26%) or BRAF (28%) mutations. KRAS and PIK3CA tended to be concurrently mutated, in a statistically and biologically significant fashion (OR = 2.97, 95% CI 1.27–7.40, *p* value = 0.0076, see Table 1).

**Table 1:** Mutual relations of investigated genes

	KRAS	NRAS	BRAF	PIK3CA
KRAS	–	–	–	–
NRAS	0.15 (0.03–0.71), 0.0065**	–	–	–
BRAF	0.15 (0.04–0.47), 0.0002***	0.47 (0.01–3.33), 0.6993	–	–
PIK3CA	2.97 (1.27–7.40), 0.0076**	2.18 (0.47–8.03), 0.2517	1.65 (0.50–4.72), 0.3930	–

Values represent odds ratios with 95% CI in parentheses, p-values are in italics if < 0.05, and highlighted with one asterisk if < 0.05, two if < 0.01, or three if < 0.001

Concomitant KRAS, BRAF, or NRAS mutations were detected in 18 of 21 tumors (85%) with a PIK3CA exon 9 mutation and in eight of 8 tumors (100%) with a PIK3CA exon 20 mutation. Moreover, we found tumors with multiple mutations in the same gene including six tumors with two KRAS mutations, one tumor with three KRAS mutations, and one tumor with two BRAF mutations. Of these, two tumors harbored two KRAS mutations in the same codon. This combination of multiple mutations in the same gene highlighted by different allelic frequency is reported in Table 2.

**Table 2:** Patients presenting with concomitant and multiple mutations in the KRAS, NRAS, BRAF and/or PIK3CA genes

Patient ID	Gene	aa substitution	AF
K22-13	KRAS	G12S	0.11
	KRAS	G12V	0.1
	PIK3CA	D725N	0.08
	PIK3CA	H1047Y	0.16
K26-13	KRAS	G12D	0.39
	KRAS	T58I	0.08
	BRAF	S467L	0.12
K60-14	KRAS	G12C	0.1
	KRAS	G13C	0.11
	KRAS	G12V	0.12
K13-14	KRAS	G12D	0.31
	KRAS	V9F	0.09
	BRAF	R603L	0.16
K41-14	KRAS	A59E	0.27
	KRAS	G12S	0.08
	NRAS	G12V	0.07
K65-13	KRAS	A146V	0.44
	BRAF	G466V	0.33
K168-13	KRAS	G12V	0.47
	KRAS	G15S	0.07
K35-13	KRAS	G12C	0.06
	KRAS	G13D	0.54
K197-13	KRAS	G13V	0.46
	NRAS	G13S	0.06
K10-14	KRAS	G12D	0.06
	BRAF	G466V	0.11
K176-13	BRAF	S467L	0.09
	BRAF	V600M	0.09

aa, amino acid; AF, allelic frequency

### Survival analyses

We collected clinical data concerning progression-free survival (PFS) and type of chemotherapy for 101 patients. Out of these, 75 underwent first-line chemotherapy. In particular, 24 patients were treated with a combination of fluorouracil-based chemotherapy and an anti-EGFR moAb (either cetuximab or panitumumab), 26 underwent a similar backbone chemotherapy regimen with anti-VEGF moAb (bevacizumab), and 25 received a chemotherapy-only first-line therapy (either single-agent capecitabine or fluorouracil or combination with irinotecan or oxaliplatin). Tumor side (simplified as left for rectal, sigmoid, and descendant; and right for ascendant or transverse) was available for 67 patients. Information concerning tumor side, mutational status, and type of chemotherapy were available for 63 patients.

**Fig. 3:**
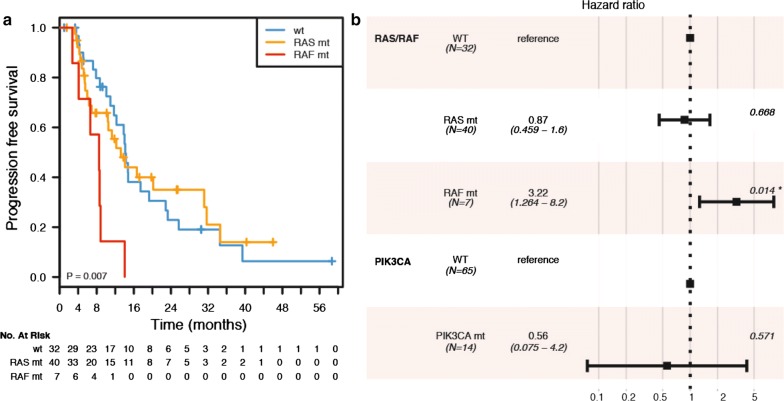
Kaplan–Meier survival analyses and forest plot according to gene mutation status. Correlation between the pathway mutations and progression-free survival (**a**). wt, wild-type; RAS mt, RAS mutated; RAF mt, RAF mutated. Forest plot of Cox proportional hazards regression with RAS/RAF mutation status and PIK3CA. mutation status (**b**). p-value < 0.05 is highlighted with one asterisk

**Fig. 4:**
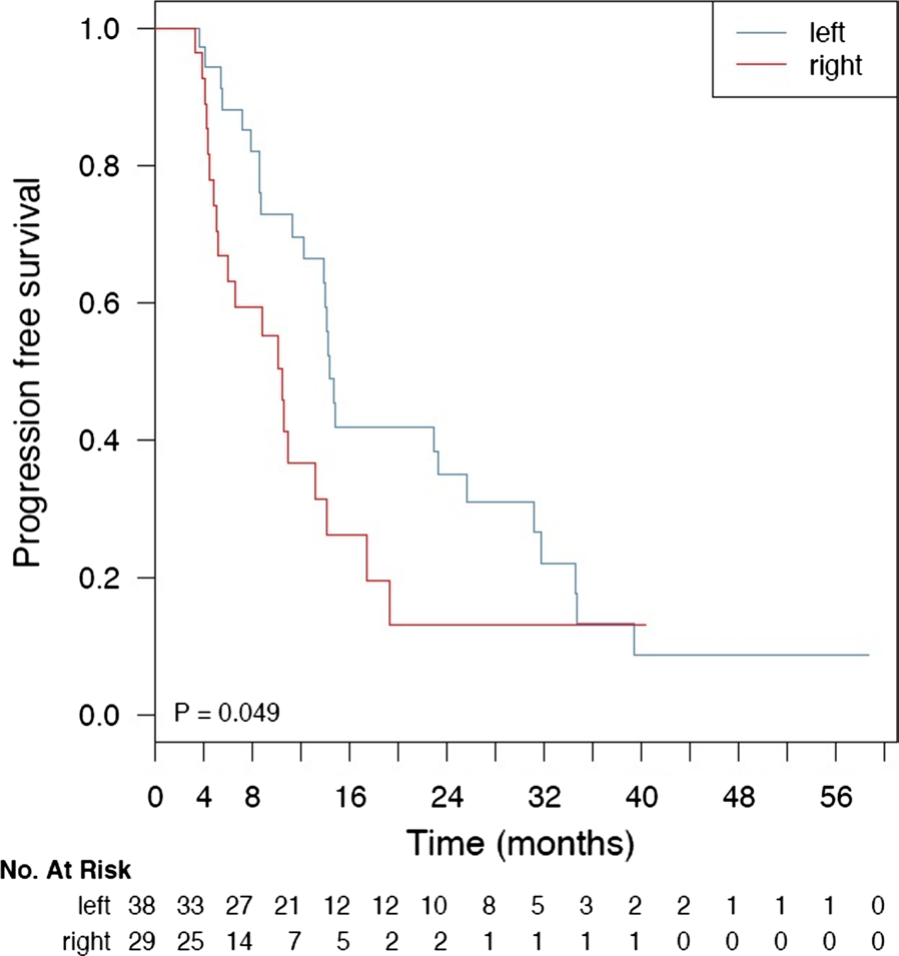
Kaplan–Meier survival analyses according to anatomical site. Correlation between tumor side and progression-free survival

By univariate analysis, BRAF-mutant patients (N = 7) had a shorter PFS compared with KRAS/NRAS mutant patients (N = 40, hazard ratio—HR = 3.58, 95% confidence interval—CI 1.48–8.66), and wild-type ones (N = 32, HR = 3.44, 95% CI 1.42–8.30) (p-value = 0.0073, logrank test, two-sided)—see Fig. 3a. The worse prognosis associated with mutated BRAF mutation was conserved by multivariable analysis, when taking into account PIK3CA status (adjusted HR = 3.22, 95% CI 1.26–8.20, p-value = 0.0142 by Cox proportional hazards regression) and the interaction of PIK3CA status with KRAS/NRAS or BRAF status (p-value for interaction = 0.4620 and 0.5870 respectively)—see Fig. 3b. Since most PIK3CA mutations co-occurred with mutations in KRAS/NRAS/BRAF (25% vs. 6%), we assessed whether PIK3CA status could stratify the subgroup of mutated patients (N = 47) into different prognostic groups, but this analysis yielded non-significant results (p-value = 0.5686, logrank test, two-sided). Since all PIK3CA exon 20 mutations co-occurred with RAS ones, we could not stratify our analyses for PIK3CA exon 9 vs. 20 mutations. Finally, we aimed at evaluating whether tumor side may have prognostic significance when considered alone or by multivariable analysis together with BRAF status, type of first-line chemotherapy, and the interaction of these two variables. Indeed, right side was associated with worse PFS by univariate statistical assessment (p-value = 0.0494, logrank test, two-sided)—see Fig. 4. Our Cox proportional hazards model was underpowered for analyses with more than 4–5 degrees of freedom, with 44 events and 9 degrees of freedom. As such, our results are of exploratory interest only. Of note, however, tumor side was the only variable independently associated with a PFS, with the direction of the effect in agreement with the univariate log-rank test. Due to the limitation in power size, we do not report formal results concerning this latest analysis.

## Discussion

In this real-world study, we characterized retrospectively 219 consecutive cases of metastatic colorectal cancer using NGS in clinical practice. We explored the association of clinico-pathological variables related to the mutational status of KRAS, NRAS, BRAF, and PIK3CA, genes that are involved in the main pathways of different solid tumors including colorectal cancer. Due to the high analytic sensitivity of NGS we were able to assess the concomitant presence of low-frequency aberrations which can be assessed only by non-standard and often cumbersome methods [18].

In our cohort, 47% of patients had a KRAS mutation (42% in exon 2, 7% in exons 3 and 4). Seven percent of patients had a NRAS mutation (3% in exon 2, 4% in exon 3). Thirteen percent of patients had a BRAF mutation, 9% had BRAF V600E mutation. These percentages do not differ from those described in previous clinical studies [19].

The negative prognostic role of BRAF V600E is known, whereas its predictive value for response to anti-EGFR therapy is more controversial, partly because of the small number of patients with this mutation described in clinical trials [6, 20, 21]. Recently, results from the SWOG S1406 trial, demonstrated that BRAF V600E had a predictive value for response to vemurafenib [22].

On the contrary, patients with BRAF mutations outside codon 600 could have different outcomes. Compared to patients with V600 BRAF mutations who require aggressive treatment [23], patients with non-V600 BRAF mutations appear to have an excellent prognosis [24]. Identification of non-V600 BRAF mutations could therefore lead to better stratification of mCRC patients with different therapeutic needs. In our cohort we identified 8 cases (3.5%) with tumors harboring a non-V600 BRAF mutation. This is a non-negligible proportion of patients considering the epidemiology of CRC, and its clinical characterization by routine NGS seems a sensible goal in the post-marketing age of anti-EGFR moAbs.

Concomitant RAS and BRAF mutations are considered to be rare. Indeed, data from the literature report a very low incidence of such event [25]. These epidemiological data rely however mostly on capillary sequencing findings (low sensitivity) or relatively low-coverage tumor exome sequencing from basic-translational studies. Here, we found four patients (1.8%) with concomitant RAS and BRAF alterations. Of interest, all four patients harbored a non-V600E mutation, which may be suggestive of either a lack of functional relevance of such funding, or, more intriguingly, of underlying peculiar biology of KRAS/non-V600E BRAF co-mutated tumors.

As reported by other authors [25], PIK3CA tends to be concurrently mutated with the other genes we studied. The most frequent association was with KRAS (10% in our cohort of patients).

The distribution of mutations according to anatomical site was in line with previous works [26]. Jensen et al. [27] described BRAF mutations as more frequent in right colon. Likewise, mutations in KRAS tended to be found more frequently in such anatomical region, although this association was not as statistically strong as the one with BRAF. Furthermore, microsatellite instability was strongly related to BRAF mutations, which is a well-established biological phenomenon driven by BRAF genetic sequence [28].

The correlation between mutations and sex in mCRC is uncertain. In the present work, we found that mutations in BRAF and KRAS were more frequently associated with female sex. Although we cannot completely exclude selection biases driving such finding, we were not able to find any obvious explanation for this correlation. If proven true in future, larger epidemiological studies, the association of specific mutations with sex in colorectal cancer would be of potential biological relevance.

By multivariate non-aprioristic approaches, we identified two distinct clinical-mutational profiles. The first was enriched in patients with BRAF mutations, right colon cancer, and advanced age, in agreement with previous findings [29, 30]. The second cluster exhibited mutations in KRAS and PIK3CA in younger women. As highlighted, this association is not clearly reported in literature, so that further analyses are warranted to corroborate it and ascertain its underlying significance.

To our knowledge, only few works investigated in detail the concomitant presence of multiple mutations in a single gene [31]. Here, we described several cases defined by this peculiar characteristic in our cohort. Although we assessed fewer genes compared to larger multipanel NGS studies [32, 33], the strength of the present work lies in its real-word nature: as far as we know this is the first report of such nature.

Hence, it is not unexpected that we could identify eight patients (3.5%) with more than one mutation in a single gene. The presence of these multiple and concomitant mutations, some of which at low allelic frequency, reflects the likely existence of subclonal populations within the tumor as an expression of intratumoral heterogeneity [34]. Currently, it is not clear whether tumor property could result in a more aggressive phenotype [35, 36]. Also, whether early identification of these subclonal populations should influence treatment strategies is a matter of active debate [37].

A major value-added of our study is the assessment of the relationship between RAS, PIK3CA, and BRAF tumor mutation status with clinical response to chemotherapy in the metastatic setting. BRAF mutation was prognostic for poor progression-free survival (PFS) by univariate and multivariate analysis, adjusted for the interaction of PIK3CA status with KRAS/NRAS or BRAF status. PIK3CA status by itself had no ability to stratify subgroups of mutated patients with different prognostic groups, as reported in previous works [38]. PIK3CA mutational co-occurrence with RAS aberrations may be among the reasons why the independent prognostic and predictive role of PIK3CA mutations in mCRC remains uncertain [39]. Indeed, we could not demonstrate that co-mutated KRAS/PIK3CA cases are endowed with different clinical consequences compared with KRAS-only mutant ones.

We did not observe significant differences for PFS in wild-type or RAS-mutated patients undergoing first-line treatment, probably due to the small number of patients included in the analysis. However, we were able to establish a negative prognostic value of tumor side irrespective of BRAF status and type of chemotherapy administered as first-line. Indeed, tumor side appeared as the only variable independently associated with PFS, as recently reported by other colleagues [40].

Since landscape works such as that of Galon et al. [41], where an immunological score with prognostic value based on lymphocyte infiltration in CRC was first described, increasing knowledge of cancer immunity has been changing tumor treatment. This phenomenon has been particularly evident in lung cancer and melanoma, with the introduction of novel immune-acting drugs [42, 43]. Currently, immunotherapy has been shown to have only modest efficacy in CRC, with possible exceptions in microsatellite-unstable tumors [44]. This modest effectiveness has been in part associated with the extreme heterogeneity of CRC based on molecular subtypes but is not completely explained by them. For example, it was described that KRAS mutation is associated with suppressed Th1/cytotoxic immunity in CRC [45], whereas another work demonstrated that pathway mutations such as those in the RAS/RAF or PI3KCA pathways are associated with different expression of tumor leucocyte fraction [46]. Hence, we suggest that in future research, somatic mutations in CRC driver genes be also investigated in association with immune-related parameters, to obtain a more precise immunobiological stratification of patients.

We are aware of the limitations of our study. For example, tumor microsatellite instability status was available for only half of our patients, and this may have contributed to the lower MSI frequency we described in our mCRC compared to other studies. Also, survival analyses have a limited statistical power due to the absence of outcome data for half of our patients. Nonetheless, we believe that a major strength of the present work lies in its real-world nature and in the inherent simplicity of the design of our panel. As such, we were able to convey biological and clinical findings that can really be applied to the general population, with relevant consequences for clinical practice. Furthermore, our NGS panel is cancer-type specific, fitted for a clinical context where there is need to maximize the cost/utility ratio of analyses for diagnostic and prognostication according to the characteristics of CRC specimens sent to our center and to the scientific evidence of specific mutations. Certainly, reagent cost and analysis turnaround time for NGS targeted panels are easy argument for extending the use of NGS to multicancer assays. However, it is our personal feeling that, outside clinical trials and cancer landscaping efforts, such extended panels may be of nontrivial use, and in certain situations could even lead to potentially detrimental effects (e.g., the difficulty in clinical interpretation and subsequent use of novel identified alterations). Broader, multicentric, more standardized approaches need to be implemented to properly assess the effective medical utility of large, multicancer NGS panels.

## Conclusions

Our present research strongly supports the routine application of NGS in the clinical practice context for the diagnosis of mutations in mCRC. NGS indeed leads to a more accurate stratification of patients benefitting from anti-EGFR treatment compared to commercially available non-NGS-based kits. Similarly, we provide evidence in clinical practice that low-frequency subclones of heterogeneous, yet already anti-EGFR resistant clones exist in primitive colorectal tumors, with strong implications for targeted treatment planning and design of future clinical trials.

## Additional file

**Additional file 1.** Additional tables and figures.

## Abbreviations

AF: allelic frequency
CRC: colorectal cancer
EGFR: epidermal growth factor receptor
dPCR: digital polymerase chain reaction
FFPE: formalin-fixed paraffin-embedded
MCA: multiple correspondence analysis
mCRC: metastatic colorectal cancer
moAbs: monoclonal antibodies
NGS: next-generation sequencing
PFS: progression-free survival
VEGF: vascular endothelial growth factor
