# Supplementary material for: Clinico-pathological associations and concomitant mutations of the RAS/RAF pathway in metastatic colorectal cancer
Source: J Transl Med. 2019 Apr 29;17:137. doi: 10.1186/s12967-019-1879-2 (PMC6489172; doi:10.1186/s12967-019-1879-2)
Supplement: Supplementary file 1 — Additional file 1. Additional tables and figures. [file 12967_2019_1879_MOESM1_ESM.docx]

**Additional File 1 – Additional tables and figures**

| **Step type** | **Time** | **Temperature (°C)** |
| --- | --- | --- |
| Hold | 10 min | 96 |
| Cycle (40 cycles) | 2 min | 56 |
| Cycle (40 cycles) | 30 s | 98 |
| Hold | 2 min | 60 |
| Hold | ∞ | 10 |

**Additional File 1 : Table S1**

**PCR-run protocol**


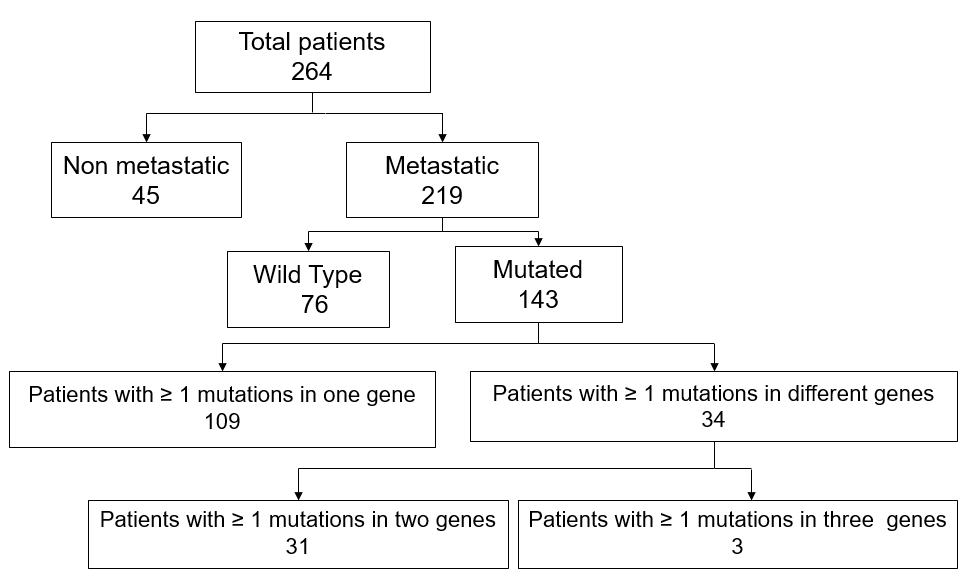


**Additional File 1 : Figure S1**

**Consort-like flow chart**

| **Characteristics** | **Number of patients** | **Percent of patients (%)** |
| --- | --- | --- |
|  |  |  |
| **Age (years)** |  |  |
| < 66 | 78 | 36 |
| 66-75 | 68 | 31 |
| > 75 | 67 | 31 |
| n.a. | 6 | < 1 |
|  |  |  |
| **Sex** |  |  |
| Male | 115 | 52 |
| Female | 104 | 48 |
| n.a. | 0 | 0 |
|  |  |  |
| **Anatomical site** |  |  |
| Right | 68 | 31 |
| Left | 151 | 69 |
| n.a. | 0 | 0 |
|  |  |  |
| **T size** |  |  |
| T1-3 | 100 | 46 |
| T4 | 56 | 25 |
| n.a. | 63 | 29 |
|  |  |  |
| **N status** |  |  |
| Negative | 24 | 11 |
| Positive | 132 | 60 |
| n.a. | 63 | 29 |
|  |  |  |
| **MSI staining** |  |  |
| Conserved | 104 | 48 |
| Lost | 9 | 4 |
| n.a. | 106 | 48 |
|  |  |  |

**Additional File 1 : Table S2**

**Patients and sample characteristics (n = 219)**

|  | **KRAS mt/wt** | **NRAS mt/wt** | **BRAF mt/wt** | **PIK3CA mt/wt** |
| --- | --- | --- | --- | --- |
|  |  |  |  |  |
| **Age** n = 213 | 0.9929 | 0.3881 | 0.2026 | 0.5193 |
| Median (IQR) – mt | 69 (63-77) | 70 (62-72) | 72 (60-83) | 69 (60-75) |
| Median (IQR) – wt | 70 (61-76) | 70 (61-77) | 70 (61-76) | 70 (62-76) |
| n.a. | 6/0 | 0/6 | 0/6 | 1/5 |
| **Sex (F vs. M)**  n = 219 | 1.81 (1.01-3.29), 0.0460 | 0.35 (0.09-1.14), 0.0847 | 2.59 (1.11-6.30), 0.0269 | 1.40 (0.64-3.12), 0.4026 |
| Female | 57 (26) / 47 (22) | 3 (1) / 101 (46) | 18 (8) / 86 (39) | 19 (9) / 85 (39) |
| Male | 47 (22) / 68 (31) | 12 (6) / 103 (47) | 10 (5) / 105 (48) | 14 (6) / 101 (46) |
| n.a. | -- | -- | -- | -- |
| **T (4 vs. 1-3)**  n = 156 | 0.71 (0.34-1.45), 0.3467 | 0.28 (0.05-1.05), 0.0592 | 1.14 (0.46-2.76), 0.7810 | 1.20 (0.47-2.97), 0.6950 |
| 1-3 | 46 (30) / 54 (35) | 12 (8) / 88 (56) | 15 (10) / 85 (54) | 16 (10) / 84 (54) |
| 4 | 23 (15) / 30 (21) | 2 (1) / 54 (35) | 11 (7) / 45 (29) | 9 (6) / 47 (30) |
| n.a. | 35 / 28 | 1 / 62 | 2 / 61 | 8 / 55 |
| **N (positive vs. negative)**  n = 156 | 1.96 (0.78- 5.18), 0.1554 | 2.73 (0.57- 26.89), 0.2316 | 2.81 (0.78-15.07), 0.1206 | 0.64 (0.21-2.30), 0.4739 |
| Negative | 9 (6) / 15 (10) | 1 (<1) / 23 (15) | 2 (1) / 22 (14) | 4 (3) / 20 (13) |
| Positive | 60 (39) / 72 (42) | 13 (8) / 119 (76) | 24 (15) / 108 (69) | 21 (14) / 111 (71) |
| n.a. | 35 / 28 | 1 / 62 | 2 / 61 | 8 / 55 |
| **Anatomical site (right vs. left)**  n = 219 | 2.56 (1.33- 5.11),  0.0048 | 1.50 (0.41- 4.84), 0.5184 | 6.70 (2.77-17.01), **<** 0.0001 | 0.46 (0.17-1.08), 0.0770 |
| Left | 67 (31) / 84 (38) | 11 (5) / 140 (64) | 11 (5) / 140 (64) | 25 (11) / 126 (58) |
| Right | 37 (17) / 31 (14) | 4 (2) / 64 (29) | 17 (8) / 51 (23) | 8 (4) / 60 (27) |
| n.a. | -- | -- | -- | -- |
| **MSI staining (lost vs. conserved)**  n = 113 | 0.61 (0.05-5.48), 0.6537 | 1.44 (0.01- 28.24), 0.8339 | 13.84 (2.79-107.43), 0.0009 | 1.02 (0.08-8.44), 0.98221 |
| Lost | 1 (<1) / 8 (7) | 0 (0) / 9 (8) | 7 (6) / 2 (2) | 1 (<1) / 8 (7) |
| Conserved | 48 (50) / 56 (43) | 8 (7) / 96 (85) | 12 (11) / 92 (81) | 13 (12) / 91 (81) |
| n.a. | 55 / 51 | 7 / 99 | 9 / 97 | 19 / 87 |

**Additional File 1 : Table S3**

**Patient characteristics in relation to gene mutational status.**

mt = mutated, wt = wild-type, IQR = interquartile range, n = number of patients with available information for a given variable, n.a. = number of patients with unavailable information for a given variable, F = female, M = male. For age as a continuous variable, P is reported, while for categorical variables, odds ratios with 95% confidence intervals, followed by P values are indicated. For each level of categorical variables, numbers with proportions of mt/wt available patients are also reported.
